# Genetic Polymorphisms Associated with Lithium Response in Bipolar Disorder: An Integrative Review and In Silico Protein–Protein Interaction Analysis

**DOI:** 10.3390/ph19030511

**Published:** 2026-03-20

**Authors:** Ovinuchi Ejiohuo, Aleksandra Szczepankiewicz

**Affiliations:** 1 Department of Psychiatric Genetics, Poznan University of Medical Sciences, Rokietnicka 8, 60-806 Poznan, Poland; 2 Molecular and Cell Biology Unit, Poznan University of Medical Sciences, 60-572 Poznan, Poland; 3 Experimental Medicine Centre, Poznan University of Medical Sciences, Rokietnicka 8, 60-806 Poznan, Poland

**Keywords:** lithium response, genetic polymorphism, bipolar disorder, in silico, protein–protein interaction

## Abstract

**Background/Objectives**: Management of bipolar disorder is marked by variability in lithium response, with responders constituting a distinct clinical subgroup. Although pharmacogenetic studies implicate polymorphisms in neuroplasticity-related genes (*BDNF*) and hypothalamic–pituitary–adrenal (HPA) axis regulators (*NR3C1*), the underlying biophysical mechanisms remain poorly characterized. This study aims to bridge this structural–mechanistic gap by quantifying the atomic-level effects of key lithium-response polymorphisms on protein–protein interaction stability and conformational dynamics. **Methods**: Variant sequences for *BDNF* rs6265 and *NR3C1* rs56149945 were generated and structurally modeled with SWISS-MODEL. Protein–protein interaction analyses focused on the BDNF–TrkB and NR3C1–FKBP5 systems. Structural alignment and conformational comparisons were performed with ChimeraX and US-align, while interaction energetics were evaluated with PRODIGY and HawkDock. Conformational flexibility was assessed using CABS-flex through RMSF analysis. **Results**: Structural validation showed acceptable model quality. Binding analyses indicated stronger interactions in the variant complexes. In the BDNF–TrkB complex, binding affinity shifted from −13.8 to −15.1 kcal/mol with an ~8.5-fold lower dissociation constant, while the NR3C1–FKBP5 variant complex shifted from −16.3 to −18.8 kcal/mol with an ~65-fold lower dissociation constant. MM/GBSA calculations supported increased stability, with binding energies changing from −61.98 to −83.91 kcal/mol (BDNF–TrkB) and from −18.88 to −31.25 kcal/mol (NR3C1–FKBP5). Structural superposition showed high conservation of global folds (pruned RMSD 0.779 Å and 0.310 Å; TM-scores 0.753 and 0.967). RMSF profiles were largely overlapping, indicating localized interface adjustments rather than global conformational changes. **Conclusions**: These findings suggest that lithium-response polymorphisms may modulate protein–protein interaction stability while preserving overall structure, providing a structural framework for exploring genetic influences on lithium treatment response.

## 1. Introduction

Lithium salts remain the gold standard and a first-line agent for the treatment of bipolar disorder (BD), effective for mood stability and its unique anti-suicidal properties [1,2,3]. However, substantial inter-individual variability in treatment response is a persistent clinical challenge, with only a subset of patients achieving full prophylactic benefit. Previous studies showed that 30–55% of BD patients receiving lithium exhibit only partial or poor response, whereas other studies suggested that about 50–60% BD patients in manic episodes do not respond well to lithium and require additional mood-stabilizing agents [4,5,6,7]. Genetic contributions to this variability are strongly supported by family-based studies demonstrating the heritability of lithium response phenotypes [8,9]. It was also suggested that patients responding well to lithium constitute a distinct subgroup of BD patients who show a different disease course and clinical profile [10]. These observations motivated the extensive investigation of genetic determinants over the past two decades [7].

Genome-wide association studies (GWAS) and targeted candidate gene analyses in BD have implicated multiple genetic loci associated with differential lithium response. The largest collaborative GWAS conducted by the International Consortium on Lithium Genetics (ConLiGen) identified four single-nucleotide polymorphisms (SNPs) on chromosome 21 (rs79663003, rs78015114, rs74795342, rs75222709) that were significantly associated with lithium response, highlighting the polygenic nature of lithium efficacy [11]. The response-associated region contained two genes for long non-coding RNAs (lncRNAs), AL157359.3 and AL157359.4, that might be potential regulators of gene expression, but functional studies are necessary to explain the biological context of these genes and their clinical utility. The identification of non-coding RNAs rather than classical protein-coding genes highlights the complexity of lithium pharmacogenetics beyond classic candidate genes. Another GWAS found an intronic variant (rs116323614) associated with *SESTD1* in lithium-responsive BD, but with current limitations for clinical use, demonstrating both the promise and the limitations of GWAS for pharmacogenetic discovery [12]. A Taiwan GWAS in Han Chinese identified intronic *GADL1* variants (rs17026688/rs17026651) that strongly predicted good response, but this has not been replicated in other ancestries [7,13,14].

Previous candidate gene studies from our research center have focused on pathways integral to neuroplasticity and neurotransmission, including variants in genes located within the brain-derived neurotrophic factor (*BDNF*), its receptor NTRK2, and FYN kinase, as well as genes within intracellular signaling cascades and glutamatergic transmission, such as *GRIN2B* (NMDA receptor) and *GRIA2* (receptor AMPA). However, many candidate gene associations fail to replicate across different ethnic populations, and individual studies often lack the power to detect small effect sizes and to account for phenotypic heterogeneity that affects how the lithium response is defined, leading to conflicting data [7,11,15,16]. More recently, genes related to stress axis regulation have also emerged in lithium genetics studies, including *NR3C1* and *FKBP5* [17]. It is important to note that no single polymorphism is currently used as a standalone clinical test for lithium response. Polygenic studies support this notion, showing that lithium response reflects the combined influence of multiple biological pathways rather than single-gene effects. Lithium-response polygenic scores (Li^+^-PGS) have shown that patients in the highest genetic risk decile have approximately 3.5-fold higher odds of a good response to lithium than those in the lowest decile, with enrichment observed in cholinergic and glutamatergic signaling pathways [18]. Similarly, pathway-specific PGS analyses indicate that increased genetic loading in acetylcholine, GABAergic signaling, calcium channel, glycogen synthase kinase (GSK), and circadian rhythm pathways predicts better treatment response, whereas stronger mitochondrial pathway loading is associated with poorer outcomes [19].

In regard to neurodevelopment and the *BDNF* gene, Rybakowski et al. 2005, for the first time, showed that the Val66Met (rs6265) polymorphism was associated with long-term lithium prophylaxis outcomes in BD patients who received lithium for 5–27 years (mean 16 years) [20]. Taking into account that the Val66Met variant causes an amino acid change, we further investigated whether *BDNF* may act in epistasis with other genes and thus influence lithium response. A study by Rybakowski et al. 2007 [21] found that serotonin transporter gene *5-HTTLPR* (short/long variant) interacts with this *BDNF* polymorphism, and that carriers of the S variant and homozygous for the Val allele were more frequently non-responders to lithium prophylaxis. A study from our group that investigated the interaction between *BDNF* variants and the *NTRK2* gene, which encodes a specific receptor for *BDNF*, confirmed an association with *BDNF* polymorphisms but found no association between *NTRK2* variants and lithium response [22].

In the context of negative findings regarding lithium action and variants in genes involved in neurodevelopment and neurotransmission pathways, we found no evidence for an association between variants in the *FYN* and *GRIN2B* genes and lithium treatment response in our group of BD patients [23,24]. Similarly, no evidence for association was found for the promoter polymorphism in the glycogen synthase kinase gene (*GSK3β*) [25]. However, genetic variation in *GSK3β* remains biologically relevant because lithium inhibits *GSK3β* activity, which is hypothesized to mediate mood stabilization [26,27,28,29]. *GSK3β* promoter SNPs, such as rs334558, have been associated with lithium efficacy in pharmacogenetic studies [30], suggesting a role in mechanisms of treatment response. Exome sequencing has also identified *AKAP11* as a high-confidence risk gene for bipolar disorder that interacts with *GSK3β* [31]. Although the finding was inconclusive, data from carriers of *AKAP11* PTV rare variants showed a trend toward better lithium response [31]. While evidence for the *CACNA1C* gene and its variants specifically modulating lithium response is inconclusive [32], previous studies suggested an association of two variants, rs2370413 and rs11062170, with clinical outcomes, including lithium response, in BD [33,34,35]. *ANK3* and its polymorphisms (rs1002442, rs10994359, rs4948418, rs10994415, rs10994397, rs10994338, rs10761482, and rs10994336) were shown to mediate lithium response in BD [36,37,38]. This was further validated in functional studies using *ANK3* p.W1989R mouse models of BD, showing that chronic lithium treatment restores inhibitory function by enhancing presynaptic GABAergic neurotransmission and reducing cortical pyramidal neuron hyperexcitability [39]. This, in turn, partially rescues defects in axon initial segment (AIS) length and neuronal structure, likely via *GSK3β* inhibition. Other SNPs associated with good lithium response included: rs6772967 (*APRG1*), rs2439523 (*SDC2*), rs11237637 (*TENM4*), rs9784453 (*GRIA2*), and rs5021331 (unidentified associated gene) [40]. The study by Squassina et al. (2011) identified rs11869731 (*ACCN1*), rs2811332 (*TMCC1*), rs1390913 (*GNPDA2*), and rs869156 (*RASSF4*) as marginally associated with lithium response in BD [41]. These findings make variant/SNP biomarkers an important component, alongside neuroimaging and peripheral biomarkers, in the investigation of lithium response in BD [42].

Stress axis regulation has also emerged in lithium genetics studies. Variants in hypothalamic–pituitary–adrenal (HPA) axis-related genes, including *FKBP5* and other glucocorticoid pathway modulators, have shown associations with lithium response, linking stress axis regulation to mood stabilization mechanisms [43,44]. Previous findings from our center showed that polymorphisms in the glucocorticoid receptor (NR3C1) gene are associated with lithium response [43]. We found that the C allele of the rs41423247 variant is associated with excellent response, whereas five GR polymorphisms (rs6198, rs6191, rs6196, rs258813, rs33388) were in strong linkage equilibrium, and the TAAGA haplotype was more prevalent in the group of partial- and non-responders. These results suggested that genetic modulation of glucocorticoid signaling may partly underlie the heterogeneity of therapeutic outcomes with lithium. Evidence strongly supports variants in other HPA-related genes, particularly *FKBP5*, and lithium response in BD. Specifically, three polymorphisms (rs1360780, rs7748266, and rs9296158) in *FKBP5* have been linked to the degree of response to lithium, with certain haplotypes influencing treatment efficacy [44]. These findings align with evidence that stress reactivity and neuroendocrine regulation are involved in mood disorder pathology and response to mood-stabilizing treatments.

Although pharmacogenetics aimed to elucidate the complexity of lithium response, neither candidate gene associations nor genome-wide signals have yet yielded specific SNPs with clinical utility as robust predictors of lithium response. Therefore, a critical gap remains in understanding the functional mechanisms by which genetic variants influence lithium’s interaction with its molecular targets. Lithium ionic form interacts with a range of protein ligands involved in signal transduction, neuroplasticity, and cellular stress responses, yet the consequences of common genetic variation on these interactions have not been explored at the molecular level. Computational (in silico) modeling offers a powerful approach to predict how SNP-induced changes in amino acid sequences or protein conformation may alter the affinity and dynamics of lithium binding to specific protein targets.

Therefore, the present study aims to integrate the findings from genetic association studies with in silico structural modeling to evaluate how DNA variants might influence the stability and dynamics of key protein-protein complexes. By characterizing the structural impact of these polymorphisms, we seek to provide a mechanistic basis for observed clinical variations in lithium treatment response. In silico structural modeling and computational tools are increasingly vital for investigating psychiatric and mental disorders, offering innovative ways to understand complex mechanisms, accelerate drug discovery, and personalize treatment, bypassing traditional time-intensive laboratory methods [45,46,47,48,49]. Based on previous genetic findings from our center, we selected two genes, one related to neurodevelopment (*BDNF*) and the other involved in the regulation of the stress axis (*NR3C1*), to assess how specific genotypes and alleles affect lithium binding affinity. We used structure-informed in silico approaches to investigate how the *BDNF* rs6265 and *NR3C1* rs56149945 polymorphisms may modulate protein structure and binding energetics relevant to lithium response. By quantifying variant-associated changes in interaction profiles, this work extends pathway-based findings by proposing plausible, structure-based mechanisms linking variants to differential lithium response. This integrative strategy has the potential to elucidate the mechanistic causes of lithium’s therapeutic heterogeneity and may also be useful for developing future pharmacogenomic biomarkers of clinical relevance.

## 2. Results and Discussion

### 2.1. Structural Quality Assessment of Variant Models

Structural validation of the in silico variant models generated by SWISS-MODEL demonstrated overall acceptable model quality. The BDNF rs6265 (Val66Met) model exhibited high structural integrity, with a MolProbity score of 1.41, no steric clashes (clash score = 0.00), and 88.98% of residues in favored Ramachandran regions, indicating excellent stereochemical geometry. The NR3C1 rs56149945 variant showed comparatively reduced structural quality, with a MolProbity score of 2.18, a non-zero clash score of 1.16, including a localized steric clash between Arg447 and His454, and 75.14% Ramachandran-favored residues, suggesting increased conformational strain relative to the BDNF variant.

**Figure 1:**
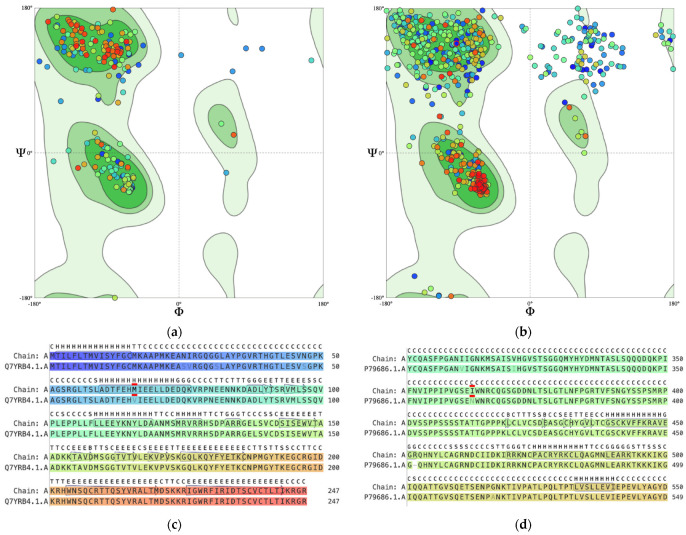
Ramachandran plot for (**a**) BDNF rs6265 and (**b**) NR3C1 rs56149945. psi—Ψ and phi—Φ (**c**) BDNF V (chain A) and SWISS-MODEL template (Q7YRB4.1.A) alignment (**d**) NR3C1 V (chain A) and SWISS-MODEL template (P79686.1.A). The color spectrum of the dots in the Ramachandran plots and alignments from purple to red represents the N to C terminus of the proteins. The red mark in c and d shows the point of the amino acid substitution. The letters at the very top of c and d (B—β bridge, E—extended β strand, G—3_10_ helix, C—coil/loop, H—α helix, I—π helix, P—polyproline II helix, S—bend, T—hydrogen bond turn) are Define Secondary Structure of Proteins (DSSP) codes that describe the physical shape of the protein backbone at that specific residue.

Almost all residues (dots) in Figure 1a fall within the dark green favored regions. suggests that the BDNF variant model is structurally sound, and that the substitution (Val66Met) has not caused a massive, unnatural distortion of the overall protein fold. The clusters are very tight, and there are very few outliers (dots in the white areas). In Figure 1b, there is a much larger spread of residues, particularly in the top-left (β-sheet) and top-right quadrants. The scattered dots between the main clusters suggest the presence of more flexible loops or intrinsically disordered regions. There is a dense cluster of red/orange dots in the α-helix region, which likely represents the core of the receptor. The scattered blue dots in the white/light green regions suggest that the variant may have more stress in its backbone or more flexible regions than the BDNF variant.

The alignment sequence for BDNF in Figure 1c shows Val66 in the SWISS-MODEL template (Q7YRB4.1.A) replaced by Met66 in Chain A. In Figure 1d for NR3C1, Asn363 in the template (P79686.1.A) is replaced by Ile363 in Chain A. This provides proof that the generated variant structures contain the amino acid substitution that makes up the variants.

### 2.2. Comparative Binding Analysis of BDNF–TrkB and NR3C1–FKBP5 Interactions

Structural docking analysis from PRODIGY indicated that the variant complexes exhibited stronger binding interactions compared with the wild-type complexes, as reflected by more negative binding affinities and lower dissociation constants (Table 1). The variants also demonstrated increased hydrophobic and mixed polar–apolar interface contacts, suggesting improved interface packing and complementarity, and hence stronger binding than the wild-type [50,51,52,53]. These changes collectively indicate enhanced protein–protein interaction stability in the variant complexes.

The Molecular Mechanics/Generalized Born Surface Area (MM/GBSA) binding energy and intermolecular contacts for the best models (model 1) are shown in Table 2. Both variants form more stable protein–protein complexes than the wild-type, as reflected by more negative binding free energies. The variant increases binding stability by ~22 kcal/mol in the BDNF–TrkB complex and by ~12 kcal/mol in the NR3C1–FKBP5 complex.

For the BDNF–TrkB complex, the dominant driver of the increased stability is a large increase in electrostatic interaction energy (ELE). Although the polar solvation penalty (GB) also increases in the variant, reflecting the energetic cost of burying additional charges at the interface, the electrostatic gain substantially outweighs this penalty, resulting in net stabilization of the complex. Such a configuration could promote a tightly bound complex in which the proteins remain strongly associated. In contrast, the stabilization observed in the NR3C1–FKBP5 complex is primarily driven by van der Waals (VDW) interactions and nonpolar surface contributions (SA) rather than electrostatics. The variant exhibits a stronger negative VDW contribution and increased nonpolar surface interaction, indicating improved hydrophobic packing at the interface.

These findings suggest that the polymorphisms induce interface remodeling via distinct mechanisms: electrostatic optimization of the BDNF–TrkB interaction and hydrophobic packing enhancement of the NR3C1–FKBP5 interaction. Such changes could influence signaling processes associated with neuroplasticity and stress-response pathways, as well as overall disease susceptibility [54,55,56,57,58], which are central to the biological functions of these systems.

TrkB initiates signaling that activates intermediate transcription factors [59,60,61,62,63,64,65,66,67], such as CREB1, which drives BDNF gene expression [68,69]. The docking analysis, which showed a higher binding affinity for the BDNF variant, provides a structural/energetics mechanism for the clinical observations reported by Ferensztajn-Rochowiak et al. (2022) [70], suggesting that the variant’s altered promoter sensitivity may facilitate lithium’s compensatory effect in BDNF-deficient genotypes. Met-carriers have lower BDNF [71,72,73,74]. There is impaired neuroplasticity in the brains of these carriers [72,75]. Because the docking shows the Met-variant has a higher affinity for TrkB, it might be more sensitive to lithium’s effect. Lithium acts as a powerful compensatory bridge. In Val/Val individuals (wild-type), the system is already full, so lithium does not change much. In Met-carriers, lithium fixes a fundamental deficit, leading to the dramatic 40% vs. 3% responder rate seen in the study by Ferensztajn-Rochowiak et al. (2022) [70].

Glucocorticoid receptor *NR3C1* polymorphisms have been associated with lithium response in bipolar individuals [3,43]. When FKBP5 binds to NR3C1, it acts as a co-chaperone, inhibiting receptor activity and reducing cortisol sensitivity, thereby impairing negative feedback of the hypothalamic–pituitary–adrenal (HPA) axis [76,77]. This glucocorticoid resistance means that the body continues to produce stress hormones, triggering mania and depression in BD (disrupted mood stability). As shown by the stronger binding, bipolar individuals with the Ile variant of the NR3C1 receptor bind their inhibitory partner (FKBP5) much more tightly than wild-type individuals. Individuals with this system might suffer from chronic neuroinflammation and low neuroplasticity [78,79,80] because their natural repair system (NR3C1) is trapped. When lithium breaks the strong binding, receptor activity increases (higher sensitivity). This is probably responsible for the higher lithium response in individuals with this polymorphism.

### 2.3. Structural Superposition and Conformational Analysis

**Figure 2:**
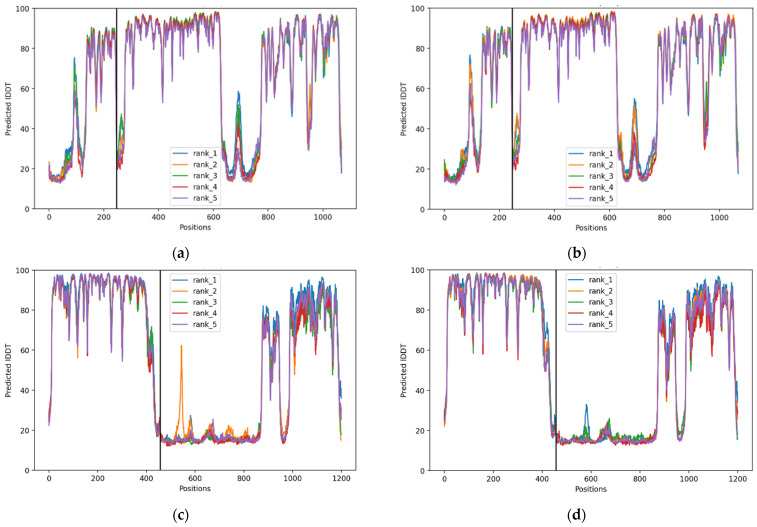
Predicted IDDT per position. (**a**) BDNF–TrkB (WT) (**b**) BDNF–TrkB (V) (**c**) NR3C1–FKBP5 (WT) (**d**) NR3C1–FKBP5 (V). The black vertical lines between residue positions represent the boundary between the two proteins in the complex.

Figure 2 shows per-residue pLDDT (Predicted Local Distance Difference Test) confidence scores generated by ChimeraX/ColabFold for the BDNF–TrkB and NR3C1–FKBP5 complexes. Across Figure 2a–d, the five predicted models (rank 1–rank 5) show very similar pLDDT patterns, indicating high reproducibility of the structural predictions. Most residues display pLDDT scores of 80–90, corresponding to high to very high structural confidence, suggesting that the core folded domains of the proteins are predicted with near-atomic reliability.

Structural superposition analysis was performed to quantify conformational differences between the compared protein complexes. The best model for the BDNF–TrkB complex, obtained from UCSF ChimeraX/ColabFold modeling, for both the wild-type and variant structures, was used. Structural alignment of the variant complex with the wild-type complex yielded a sequence alignment score of 4266.3. The root-mean-square deviation (RMSD) calculated over 471 pruned atom pairs was 0.779 Å, indicating a high degree of structural similarity across the well-aligned core regions of the complex, suggesting that the overall backbone architecture and global fold of the BDNF–TrkB complex remain highly conserved despite the presence of the polymorphism.

**Figure 3:**
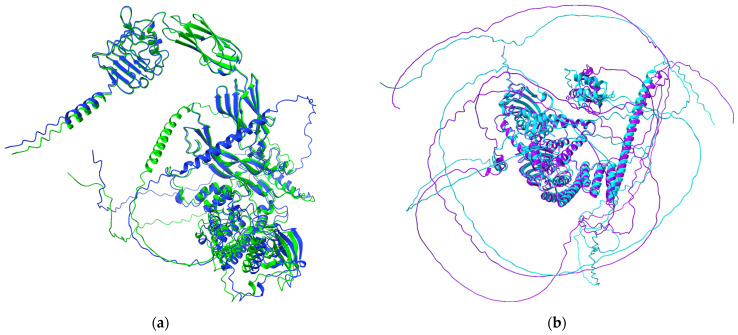
Structural analysis of the BDNF and NR3C1 model. (**a**) complex of BDNF–TrkB (WT)– blue and BDNF–TrkB (V)—green (**b**) complex of NR3C1–FKBP5 (WT)—purple and NR3C1–FKBP5 (V)—blue.

When RMSD was calculated across all 822 atom pairs, including flexible and poorly aligned regions, the value increased to 12.268 Å. This higher global RMSD reflects structural variability in peripheral or flexible regions rather than changes in the conserved structural core. These results indicate that while the global structure of the BDNF–TrkB complex is largely preserved (Figure 3a), the variant may introduce localized conformational adjustments at the protein–protein interaction interface, which could contribute to differences in binding affinity or interaction stability.

For the NR3C1–FKBP5 complex, the best models generated with UCSF ChimeraX/ColabFold were also used. Alignment of the variant complex with the wild-type produced a sequence alignment score of 3683.7. The RMSD calculated over 219 pruned atom pairs was 0.310 Å, indicating very high structural similarity within the conserved core of the complex. When all 743 atom pairs were considered, the RMSD increased to 36.428 Å, reflecting variability in flexible or peripheral regions rather than disruption of the overall protein fold. Similar to the BDNF–TrkB complex, these results suggest that the NR3C1 variant does not alter the global architecture of the complex (Figure 3b) but may introduce localized conformational adjustments that could influence interaction stability.

The TM-score from the US-align analysis is a measure of the overall structural similarity between the wild-type and the variant complexes for BDNF-TrkB and NR3C1-FKBP5. For BDNF-TrkB, TM-score = 0.753, RMSD = 2.74 Å, aligned residues = 207/247, indicating that the variant largely preserves the wild-type fold, though small local deviations exist. For NR3C1-FKBP5, TM-score = 0.967, RMSD = 1.29 Å, aligned residues = 448/457, indicating an almost identical variant to the wild-type in overall fold. These are similar and validated findings from the alignment analysis in ChimeraX, supporting the hypothesis that the variants may alter protein–protein interaction dynamics without disrupting the overall protein fold, with implications for lithium response.

**Figure 4:**
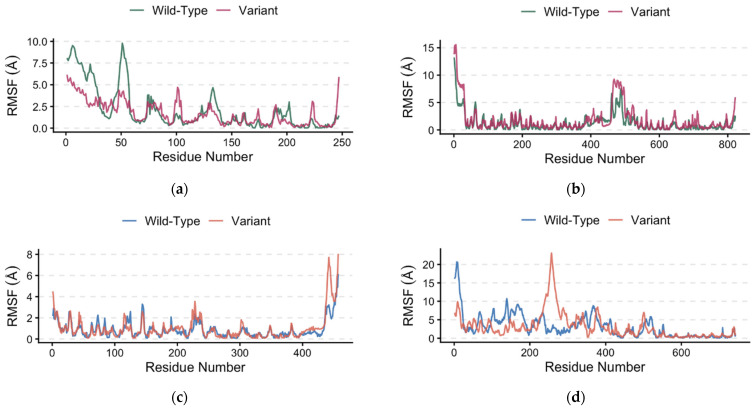
Root-mean-square fluctuation (RMSF) of the BDNF and NR3C1 model. (**a**) BDNF–TrkB chain A (**b**) BDNF–TrkB chain B (**c**) NR3C1–FKBP5 chain A (**d**) NR3C1–FKBP5 chain B.

The root-mean-square fluctuation (RMSF) profiles of the wild-type and variants show highly overlapping fluctuation patterns across most residues (Figure 4a–d), indicating that the polymorphisms do not substantially alter the global flexibility of the protein backbone. Minor deviations were confined to localized regions, suggesting that the observed differences in binding affinity are likely driven by subtle interface-level interactions, as highlighted in Table 1, rather than by large-scale conformational changes. Therefore, while global protein flexibility remains largely unchanged, minor polymorphism-induced conformational shifts as observed in our finding may influence interaction dynamics and contribute to variability in lithium drug sensitivity and response. Evidence that small structural and energetic changes from polymorphisms can influence drug response is well documented [81,82,83].

Based on these analyses and findings, we propose a double-hit structural model of lithium response, in which neuroplasticity (BDNF–TrkB) and stress-regulation (NR3C1–FKBP5) pathways are functionally constrained by high-affinity protein sequestration rather than protein loss. In this bottlenecked state, characterized by shifts in local interfaces but stable global conformational, lithium acts downstream on shared regulatory nodes, such as glycogen synthase kinase-3β (GSK-3β) and the cyclic adenosine monophosphate (cAMP) signaling pathway [84,85,86,87,88,89], to bypass structural stalls and restore pathway function. This mechanistic framework may explain why individuals with preserved but sequestered signaling scaffolds can exhibit marked lithium responsiveness, translating molecular release into superior clinical outcomes.

It is important to note that lithium response in BD is a complex, multifactorial phenotype shaped by the combined effects of multiple genes/genetic variants, biological pathways, and environmental influences, with biomarkers and clinical tools still lacking [7,90,91,92]. No single gene or polymorphism is sufficient to determine treatment response or outcome. Accordingly, the findings of this study represent a focused mechanistic exploration of a limited subset of functionally relevant polymorphisms, aimed at elucidating one plausible structural pathway through which genetic variation may modulate lithium responsiveness. These results should therefore be interpreted as contributing to, rather than exhaustively defining, the broader genetic architecture underlying lithium treatment response.

## 3. Materials and Methods

### 3.1. Sequence Retrieval and Variant Generation

The amino acid sequence of human brain-derived neurotrophic factor (BDNF; UniProt accession P23560) (accessed on 11 February 2026) was retrieved in FASTA format from UniProt (https://www.uniprot.org/). The rs6265 single-nucleotide polymorphism (Val66Met) was identified using the ProtVar interface (accessed on 11 February 2026) and modeled by manually editing the wild-type FASTA sequence to substitute valine (V) with methionine (M) at position 66, generating the BDNF V66M variant sequence. The rs6265 SNP is a missense mutation located within the protein-coding region (exon) [93,94]. Similarly, the full-length FASTA sequence of the human glucocorticoid receptor (NR3C1) was obtained from UniProt. The rs56149945 polymorphism was also identified using the ProtVar interface, and the corresponding amino-acid substitution (asparagine to isoleucine at position 363) was introduced into the wild-type sequence to generate the variant model. The rs56149945 SNP is a missense mutation located in the coding region (exon 2) of the *NR3C1* gene [95]. The BDNF/NT-3 growth factor receptor (TrkB—tropomyosin receptor kinase B) canonical FASTA sequence of the isoform Q16620-1 was retrieved from Uniprot and modeled using the SWISS-MODEL workflow (accessed on 12 March 2026). The Protein Data Bank (PDB) structure of FKBP5 was obtained from AlphaFold (https://alphafold.ebi.ac.uk/) (accessed on 10 February 2026).

### 3.2. Structural Modeling of Wild-Type and Variant Proteins

Predicted three-dimensional structures of the wild-type BDNF and NR3C1 proteins were obtained from AlphaFold. Variant-specific structural annotations for BDNF rs6265 were verified using the ProtVar platform (https://www.ebi.ac.uk/ProtVar/) [96] (accessed on 11 February 2026) to ensure positional and structural consistency. For both *BDNF* rs6265 and *NR3C1* rs56149945, variant protein structures were generated using the SWISS-MODEL server (https://swissmodel.expasy.org/) [97,98,99,100] (accessed on 11 February 2026) with its default settings and template library, and the corresponding PDB files were downloaded. The best-scoring models were selected based on sequence identity, model coverage, and global quality metrics, and their quality was validated using the derived Ramachandran plots. The Ramachandran analysis aims to validate that both variants occupy favorable conformational spaces, ensuring that the calculated docking affinities are derived from energetically plausible protein architectures [101]. Alignment images showing the amino acid substitutions were also generated.

### 3.3. Protein–Protein Binding Analysis

Binding energetics and intramolecular contacts were characterized using the PROtein binding energy (PRODIGY) prediction web server [102,103] (accessed on 12 March 2026). The PDBs of the best models for both the wild-type and variant complexes, generated by ChimeraX/Google ColabFold, were uploaded to the web server using the default settings. Binding affinity scores were compared between wild-type and variant complexes to assess the impact of the rs6265 and rs56149945 polymorphisms on molecular interaction patterns and binding affinity. Differences in binding affinity were interpreted as indicative of potential structural mechanisms underlying altered lithium response.

Molecular Mechanism/Generalized Born Surface Area (MM/GBSA) was used to assess binding affinity (ΔG) using the HawkDock version 2 webserver default settings [104,105,106,107,108,109,110]. Both wild-type and variant forms of BDNF and NR3C1 were independently docked against their respective interacting partners (TrkB for BDNF and FKBP5 for NR3C1) using their AlphaFold and Swiss-Model PDB structures. The best models from the top 10 predictions, containing electrostatic potential, Van der Waals potential, and polar-solution free energy information, were selected and compared, providing validation of the PRODIGY result.

### 3.4. Structural Modeling and Alignment Analysis

UCSF ChimeraX version 1.11 with embedded Google ColabFold [111,112] support was used to model and analyze structural interactions between wild-type and variant protein models using their sequences, which generated a “best model” PDB file. pLDDT plots were used to visualize the accuracy of the generated model complexes. Structural alignment was performed using the MatchMaker algorithm. Following alignment, structural, conformational, and visual analyses were conducted in ChimeraX to assess backbone deviations, conserved core regions, and local conformational differences, thereby validating the observed structural effects between the wild-type and variant models.

Structural similarity between the wild-type and variant complexes BDNF–TrkB and NR3C1–FKBP5 was assessed using the US-align (Universal Structural alignment) webserver [113,114], a structure alignment tool that extends TM-align [115] for pairwise and multiple alignments of proteins, RNAs, and DNAs. US-align generates optimal superpositions by maximizing the TM-score, which ranges from 0 to 1, where 1 indicates identical structures and scores ≥0.5 indicate shared global topology.

### 3.5. Protein Flexibility Analysis

Protein flexibility of the wild-type and variant structures was evaluated using the CABS-flex 2.0 web server, which performs fast coarse-grained simulations of protein dynamics and flexibility [116,117,118,119,120]. The best models for the wild-types and variants generated with UCSF ChimeraX and Google ColabFold were submitted to the server and simulated under identical conditions. Default Cα distance restraints generated by the CABS model were applied, with protein rigidity set to 1.0. Secondary structure restraints were defined as ss2, 3, 3.8, 8.0, while the global Cα and side-chain restraint weights were both set to 1.0. Each simulation consisted of 50 Monte Carlo cycles with trajectories recorded every 50 cycles. The temperature was maintained at 1.40 throughout the simulation, and a random number generator seed of 5613 was used to ensure reproducibility. Following the simulations, root-mean-square fluctuation (RMSF) values for the best model (model 1) from the top 10 predictions were used to generate a plot using R version 4.4.2 (Supplementary Data).

## 4. Conclusions

This study identifies a distinct structural signature in lithium response, in which specific genetic polymorphisms cause abnormal trapping of neuroplasticity and stress-response proteins. Although the overall protein structures remain intact, these variants induce small conformational shifts and energetics that lock the BDNF–TrkB and NR3C1–FKBP5 complexes into stalled, high-affinity states that impair normal signaling and become highly sensitive to lithium therapy.

These polymorphisms create functional bottlenecks rather than a complete loss of function. Lithium appears uniquely effective because it bypasses or destabilizes these bottlenecks, restoring downstream signaling without correcting the underlying genetic variant. As such, genetic polymorphisms in lithium therapy define biologically distinct responder subtypes and provide a mechanistic explanation for lithium’s selective efficacy, advancing pharmacogenetics from association toward structural causality.

Future work should validate the stability of these stalled conformations using molecular dynamics simulations and other experiments. The proposed double-hit model supports a personalized medicine framework in which structural metrics, such as RMSD and binding energy, could serve as biomarkers to identify lithium responders or to guide the development of therapies that mimic lithium’s rescue effect in non-responders.

## Figures and Tables

**Table 1:** Binding affinity (ΔG) and dissociation constant—Kd (M) prediction.

	BDNF–TrkB (WT)	BDNF–TrkB (V)	Difference/Significance	NR3C1–FKBP5 (WT)	NR3C1–FKBP5 (V)	Difference/Significance
ΔG (kcal/mol)	−13.8	−15.1	−1.3 shift towards higher stability	−16.3	−18.8	−2.5 shift towards higher stability
Kd (M) at 25 °C	7.3 × 10^−11^	8.6 × 10^−12^	~8.5× stronger binding	1.1 × 10^−12^	1.7 × 10^−14^	~65× stronger binding
ICs charged-charged	14	11	Reduction in electrostatic repulsion	19	18	Reduction in charged interactions
ICs charged-polar	12	12	No change	15	20	Increased electrostatic–polar interactions
ICs charged-apolar	25	38	Increase in interface complementarity	32	46	Increase interface complementarity
ICs polar-polar	4	5	Increased hydrogen-bond potential	3	5	Increased hydrogen-bond potential
ICs polar-apolar	25	27	Slight increased mixed polar–hydrophobic contacts	32	39	Increased mixed polar–hydrophobic contacts
ICs apolar-apolar	29	35	Increase in hydrophobic packing	27	39	Increase in hydrophobic packing

Number of Interfacial Contacts—ICs.

**Table 2:** MM/GBSA binding free energy analysis of wild-type and variant BDNF–TrkB and NR3C1–FKBP5 complexes.

	BDNF–TrkB (WT)	BDNF–TrkB (V)	NR3C1–FKBP5 (WT)	NR3C1–FKBP5 (V)
Total binding free energy—ΔG (kcal/mol)	−61.98	−83.91	−18.88	−31.25
VDW	−148	−85.77	−57.76	−69.78
ELE	−1039.61	−1235.66	−738.60	−702.65
GB	1144.03	1251.80	785.42	751.1
SA	−18.40	−14.27	−7.95	−9.92

## Data Availability

The original contributions presented in this study are included in the article/Supplementary Material. Further inquiries can be directed to the corresponding authors.

## Supplementary Materials

The following supporting information can be downloaded at: https://www.mdpi.com/article/10.3390/ph19030511/s1.
