# Supplementary material for: Genetic Polymorphisms Associated with Lithium Response in Bipolar Disorder: An Integrative Review and In Silico Protein–Protein Interaction Analysis
Source: Pharmaceuticals (Basel). 2026 Mar 20;19(3):511. doi: 10.3390/ph19030511 (PMC13028772; doi:10.3390/ph19030511)
Supplement: Supplementary file 1 [file pharmaceuticals-19-00511-s001.zip › pharmaceuticals-4201220-supplementary.pdf]

RMSF Plot for NR3C1 Wild Type (W) and Variant (V)

```
library(ggplot2)
library(gridExtra)

rmsf_V <- read.csv("Desktop/LitGen/rmsf_NR3C1_V.csv", header = FALSE,
          col.names = c("Residue", "Chain", "RMSF"))
rmsf_W <- read.csv("Desktop/LitGen/rmsf_NR3C1_W.csv", header = FALSE,
          col.names = c("Residue", "Chain", "RMSF"))

rmsf_V$Type <- "Variant"
rmsf_W$Type <- "Wild-Type"

rmsf_all <- rbind(rmsf_W, rmsf_V)
rmsf_all$Type <- factor(rmsf_all$Type, levels = c("Wild-Type", "Variant"))

chain_A <- rmsf_all[rmsf_all$Chain == "A", ]
chain_B <- rmsf_all[rmsf_all$Chain == "B", ]

plot_chain <- function(data, chain_label) {
  ggplot(data, aes(x = Residue, y = RMSF, color = Type, group = Type)) +
    geom_line(linewidth = 0.7, alpha = 0.85) +
    scale_color_manual(
      values = c("Wild-Type" = "#2166AC", "Variant" = "#D6604D")
    ) +
    labs(
      title = paste("RMSF of NR3C1 — Chain", chain_label),
      x = "Residue Number",
      y = expression(RMSF ~ (Å)),
      color = NULL
    ) +
    theme_classic(base_size = 13) +
    theme(
      plot.title = element_text(hjust = 0.5, face = "bold"),
      legend.position = "top",
      legend.text = element_text(size = 12),
      axis.line = element_line(color = "black"),
      panel.grid.major.y = element_line(color = "grey90", linetype = "dashed")
    )
}

p_A <- plot_chain(chain_A, "A")
p_B <- plot_chain(chain_B, "B")
```

```
grid.arrange(p_A, p_B, nrow = 2)
```

RMSF Plot for BDNF Wild Type (W) and Variant (V)

```
rmsf_BV <- read.csv("Desktop/LitGen/rmsf_BDNF_TrkB_V.csv", header = FALSE,
          col.names = c("Residue", "Chain", "RMSF"))
rmsf_BW <- read.csv("Desktop/LitGen/rmsf_BDNF_TrkB_W.csv", header = FALSE,
          col.names = c("Residue", "Chain", "RMSF"))

rmsf_BV$Type <- "Variant"
rmsf_BW$Type <- "Wild-Type"

rmsf_all <- rbind(rmsf_BW, rmsf_BV)
rmsf_all$Type <- factor(rmsf_all$Type, levels = c("Wild-Type", "Variant"))

chain_A <- rmsf_all[rmsf_all$Chain == "A", ]
chain_B <- rmsf_all[rmsf_all$Chain == "B", ]

plot_chain <- function(data, chain_label) {
  ggplot(data, aes(x = Residue, y = RMSF, color = Type, group = Type)) +
    geom_line(linewidth = 0.7, alpha = 0.85) +
    scale_color_manual(
      values = c("Wild-Type" = "#21664D", "Variant" = "maroon")
    ) +
    labs(
      title = paste("RMSF of BDNF — Chain", chain_label),
      x = "Residue Number",
      y = expression(RMSF ~ (Å)),
      color = NULL
    ) +
    theme_classic(base_size = 13) +
    theme(
      plot.title = element_text(hjust = 0.5, face = "bold"),
      legend.position = "top",
      legend.text = element_text(size = 12),
      axis.line = element_line(color = "black"),
      panel.grid.major.y = element_line(color = "grey90", linetype = "dashed")
    )
}
```

```
p_A <- plot_chain(chain_A, "A")
p_B <- plot_chain(chain_B, "B")

grid.arrange(p_A, p_B, nrow = 2)
```
